# Primary clear cell adenocarcinoma of the rectovaginal septum: A rare case report

**DOI:** 10.1097/MD.0000000000033285

**Published:** 2023-03-17

**Authors:** Zhifang Li, Guiju Zhou, Shuqiang Duan, Qing Li

**Affiliations:** a Obstetrics and Gynecology, Anqing Municipal Hospital, Anqing, China; b Obstetrics and Gynecology, The Second Affiliated Hospital of Anhui Medical University, Hefei, China.

**Keywords:** case report, primary clear cell adenocarcinoma, rectovaginal septum

## Abstract

Primary clear cell adenocarcinoma of the rectovaginal septum is a very rare event.

**Patient Concerns:**

We reported a case of a 55-year-old woman diagnosed with a lump in the vaginal rectal septum after undergoing hysterectomy with bilateral salpingo-oophorectomy in 2017, who was admitted to our department due to vaginal bleeding. Magnetic resonance imaging of the pelvis indicated the vaginal rectal space cystic and solid mass about 110 mm × 100 mm × 140 mm in size.

**Diagnosis:**

The pathological diagnosis of postoperative was clear cell adenocarcinoma.

**Interventions:**

Abdominal laparotomy showed a solid block of the vaginal rectal septum. Surgery was performed to reduce the tumor.

**Outcomes:**

This patient received 8 courses of combined chemotherapy courses after surgery for the residual lesion and achieved a complete response.

**Lessons:**

Due to the rare observation of the growth pattern, the cell morphology and immune phenotype are not specific, and clinical and pathological diagnosis is difficult. Introducing the diagnosis and treatment of this case and reviewing the literature provide a relevant reference for clinicians identification and diagnosis and treatment of this rare case.

## 1. Introduction

Women’s reproductive tract clear cell carcinoma (CCC) can occur in the ovarian, endometrium, cervical-vaginal area, and other parts of the pelvic cavity, such as the peritoneum and diaphragm.^[1,2]^ Yet, the CCC in the vaginal rectal septum is extremely rare,^[3]^ and, so far, only 2 cases have been reported.^[3,4]^ Tumor resection combined with chemotherapy is considered the main treatment for CCC in the vaginal rectal septum. Herein, we reported a primary vaginal rectal septum CCC case that reappeared 4 years after hysterectomy with bilateral salpingo-oophorectomy. We discussed the diagnostic standards and potential difficulties in the diagnosis and performed a literature review based on this unusual related event.

## 2. Case report

Institutional review board/ethics committee approval was obtained from the Institutional Review Board of the Anqing Municipal Hospital Medical Ethics (2022) No. 45. Written informed consent for publication obtained from the patients.

Our 43 years old woman was diagnosed with breast cancer in December 2009. Postoperative pathology suggested infiltrated duct cancer. Immunohistochemical staining results were: positive estrogen receptor (ER), negative progesterone receptors (PR), and primary cancer gene HER-2 (C-ERB-2) +++. The patient was treated with surgery combined with a TAC scheme (Docetaxel + EpirubicinHydrochloridefor + Cyclophosphamide), chemotherapy for 6 cycles, and endocrine therapy for 5 years (4 years of tamoxifen and 1 year of letrozole). Menstrual bleeding was not seen after the first course of chemotherapy; the last menstrual period was recorded on January 20, 2010.

On August 15, 2017, a right ovarian cystic mass (90*110 mm) was detected, and a transabdominal pelvic adhesion release, off-sleeve intrafascial hysterectomy with bilateral salpingo-oophorectomy was performed. Pathological examinations suggested that benign endometriotic cyst on the right ovary.

**Figure 1.**
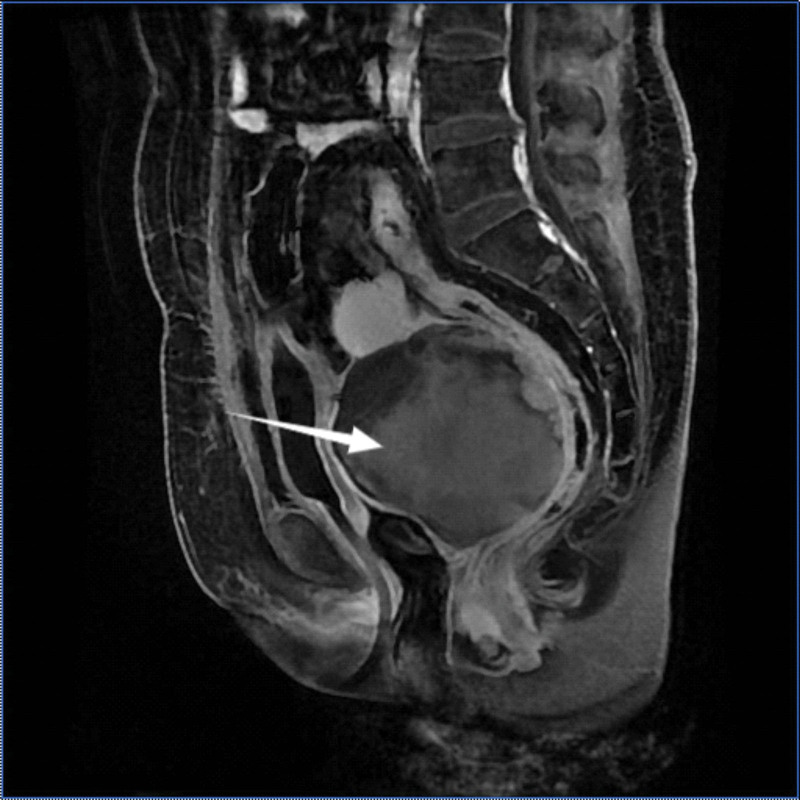
Pelvic MRI plain scan + enhanced: cystic and solid mass in the vaginal rectal space of some 11 cm × 10 cm × 14 cm in size; the signal was not uniform, the edge of TIWI was mainly iso-low signal, and patchy high signal could be seen in it. T2WI and T2 lipid-suppressed edge showed high signal, and iso- and slightly high signal were seen in them. MRI = magnetic resonance imaging.

On June 8, 2021, the patient was admitted to the Department of Anorectal Surgery, Anqing Municipal Hospital, Anhui Province, due to “blood in the stool for 1 day.” On June 9, a large quantity of vaginal bleeding (about 1000 mL) was observed; thus, the lump rupture with bleeding was considered. It was difficult to insert a vaginal speculum during the gynecological examination, and only an area 1 to 2 cm below the vagina could be exposed. Trimanual examination showed that the rectovaginal septum was almost completely occupied by a stable mass of large capacity. In addition, multiple hemorrhoids could not be returned to the anus. Blood routine analyses were: HB124G/L (94g/L after bleeding), CA125 elevated 420.43 u/mL. Sigmoidoscopy showed mixed hemorrhoids. Pelvic Magnetic resonance imaging indicated that the vaginal rectal gaps were solid, and the range of complex cysts (the source of the endometrium) was considered in combination with the medical history (Fig. 1).

On June 17, 2021, a deep mass biopsy of the rectovaginal septum was performed under vaginal color Doppler ultrasound monitoring. The biopsy results suggested bleeding necrosis and inflammatory exudate. On June 28, 2021, cytoreduction surgery was performed, during which it was found that the bottom of the basin was completely closed. After separating the adhesion junction, a solid cystic mass between the posterior vaginal wall and the rectum was seen (approx. 10 cm*8 cm*7 cm in size), with dense adhesions to the anterior wall of the rectum. During the operation, the mass was ruptured, and a large amount of dark brown bloody fluid was seen in the cyst. After the fluid in the cyst was drained, some solid tissues of fish-like necrosis were seen, accompanied by a foul smell. Fast pathological report showed a malignant epithelial tumor, which needed to be confirmed by conventional and enzyme assay.

**Figure 2.**
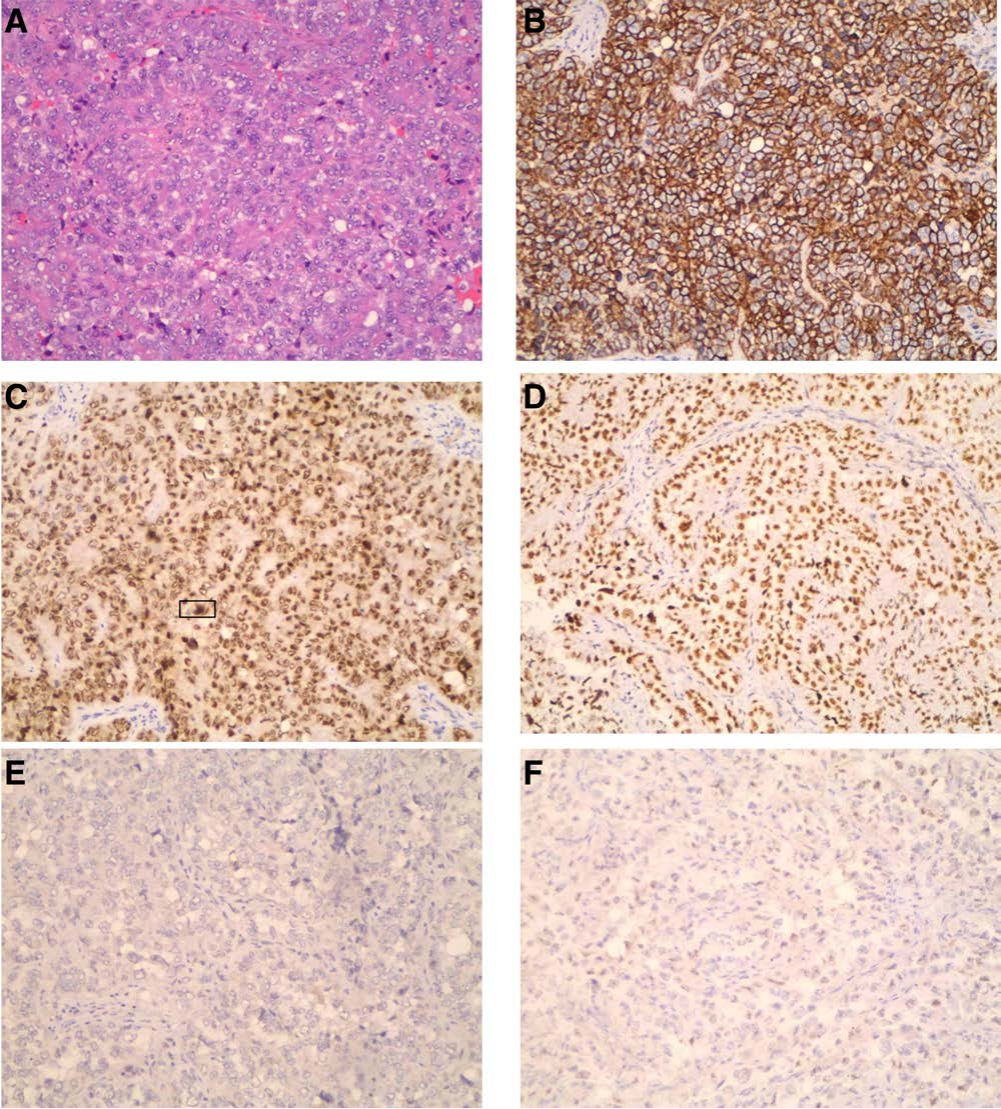
Histopathological features of the specimen (HEX100). (A) The tumor cells were arranged in solid, eosinophilic cytoplasm. (B) The tumor cells expressed CK7. (C) The tumor cells expressed PAX8. (D) The tumor cells diffusely expressed P53. (E) The tumor cells with negative WT-1. (F) Tumor cells with negative GATA3.

On July 7, 2021, immunohistochemistry confirmed that rectovaginal septum mass was a high-grade serous adenocarcinoma accompanied by wide necrosis. Immunohistochemical data were: CK7 (+), PAX8 (+), ER (−), PR (−), P53 (mutant expression), WT-1 (−), Vimentin (−), NapsinA (−), Ki -67 (~90%+), GATA3 (−), GCDFP-15 (−) (Fig. 2). Due to the unidentified source of the tumor, the Department of Pathology Department of the Cancer Hospital Affiliated with Fudan University attended the discussion on August 19. The final diagnosis was CCC. Immunization group PAX8 (+), HNF-1β (+), Napsina (−), P53 (+++), ER (−), PR (−), Vimentin (−), P16 (+), PTEN (+), WT-(−), Ki-67 (25%+); BRCA1/2 was negative.

The follow up ended on November 21, 2022. The patient received 8 courses of combined chemotherapy (nab-paclitaxel + carboplatin; bevacizumab was added in the third course), and the CA125 was normal in the third course of chemotherapy. At present, bevacizumab is used as the first-line maintenance treatment, and this patient achieved a complete response.

## 3. Discussion

Primary clear cell adenocarcinoma of the rectovaginal septum is a very rare tumor. Most cases are found with low to medium-level adenocarcinoma; however, there are also reports of clear cellular, papillary slurry, glandular, interstitial sarcoma, and cancer sarcoma histology.^[3,5–7]^ So far, only 2 cases of vaginal rectal CCC have been reported since 1978. Due to the particularity of the tumor, there are no typical symptoms in the early stage. In this case, it was only discovered when spontaneous vaginal ruptures and bleeding appeared. It is also worth noting that our patient had a history of 2 operations. No clear residual ovarian tissue or endometriosis (EM) tissue was found around the tumor intraoperative pathological diagnosis which could not rule out breast cancer metastasis.

The diagnosis of primary rectovaginal septum CCC should be performed based on the growth of the tumor and immunohistochemical staining. The positive expression of PAX8 indicates that tumors are derived from the Mullerian Duct epithelial tissue, which includes high-grade serous carcinoma, endometrial-like cancer, and CCC.^[8]^ In terms of histology, peritoneal serous carcinoma should be first considered, especially in patients with a history of personal breast cancer.^[9]^ HNF-1β has a high specific specificity for the diagnosis of ovarian CCC.^[2]^ The joint applications of immunohistochemical markers WT-1, Napsina, and HNF1-β have high specificity in distinguishing high-grade serous carcinoma and CCC. WT-1negative Napsina, and HNF1-β positive indicate CCC. Given that patients have a history of breast cancer, 2 breasts specific markers, GATA3 (−), GCDFP-15 (−), and ER (−), PR (−), combined with the pathological form of patients with primary breast tumors and hormone receptor status, the recurrence and metastasis of breast cancer was excluded.

CCC is associated with EM; arising from EM in 50% to 70% of cases.^[10]^ At present, the most accepted theory about the origin of peritoneal CCC is the vicious transformation of the Müllerian epithelialization and EM. However, only 1.6% of cases occur outside of the ovary.^[9]^ The body cavity of the female peritoneal surface has the potential to differentiate the Müllerian tube. Total hysterectomy and bilateral salpingo-oophorectomy have been performed for this patient, excluding the possibility of primary uterine and ovarian CCC. However, the patient has a 5-year history of endocrine therapy, which can activate the underlying premenopausal EM lesions, so the malignant transformation of EM of the vaginal rectal septum should not be excluded.^[11,12]^ The isolated lump in the abdominal cavity was limited to the rectovaginal septum, and there was no planting or metastasis in the pelvic or abdominal cavity. The CCC of the second Müllerian system had multiple characteristic diagnostic elements of the second Mülleriansystem tumor, after which we comprehensively considered the diagnosis. As this is a rare disease, there is a lack of systematic clinical data for retrospective research. CCC has a poor prognosis. Cytoreductive surgery is the main treatment method for this tumor.^[13]^ The chemotherapy scheme is the first choice for platinum and paclitaxel drugs, and the treatment course is not shorter than in ovarian cancer. The efficacy monitoring can be achieved by measuring serum CA125 levels.

In summary, due to its clinical and histological characteristics of CCC originating from the second Müllerian system is not specific, and the selection of immunohistochemical chemical indicators may be challenging, on account of the rarity of the general growth pattern. Clinical knowledge of the disease is insufficient. Therefore, it is difficult to argue that the tumor is the source of the second Müllerian system before and after surgery. For a clinician or a pathologist, this case represents a challenge of diagnosis as it is an extremely rare case.

In the future, more attention should be paid when establishing a diagnosis of this rare disease: if a tumor in the surgical-pathological diagnosis cannot be clearly identified, it is necessary to combine it with immunohistochemical staining, broad-spectrum cancer markers + organ-related markers + other mixed/complex markers for diagnosis. This report provides new evidence and support for the diagnosis of the tumors. Also, discoveries of new biomarkers and future treatment strategies are needed to improve local control and reduce the mortality of these patients.

## Author contributions

**Conceptualization:** Zhifang Li.

**Data curation:** Zhifang Li, Guiju Zhou, Shuqiang Duan.

**Formal analysis:** Guiju Zhou, Shuqiang Duan.

**Investigation:** Shuqiang Duan.

**Methodology:** Qing Li.

**Writing – original draft:** Zhifang Li.

**Writing – review & editing:** Qing Li.
